# Cold plasma for enhanced water purification

**DOI:** 10.1093/sumbio/qvae032

**Published:** 2024-11-14

**Authors:** Jordanne-Amee Maybin, Laura A McClenaghan, Brendan F Gilmore, Thomas P Thompson

**Affiliations:** School of Pharmacy, Queen’s University Belfast, Medical Biology Centre, 97 Lisburn Road, Belfast BT9 7BL, UK; School of Pharmacy, Queen’s University Belfast, Medical Biology Centre, 97 Lisburn Road, Belfast BT9 7BL, UK; School of Pharmacy, Queen’s University Belfast, Medical Biology Centre, 97 Lisburn Road, Belfast BT9 7BL, UK; School of Pharmacy, Queen’s University Belfast, Medical Biology Centre, 97 Lisburn Road, Belfast BT9 7BL, UK

**Keywords:** cold atmospheric plasma, water sanitation, Sustainable Development Goals, water treatment technologies, biofilm

## Abstract

Access to clean water remains a critical global challenge, with traditional water treatment methods often failing to eliminate complex pollutants such as pharmaceuticals, dyes, and antibiotics. Cold atmospheric plasma technology offers a promising solution by generating reactive oxygen and nitrogen species at room temperature, effectively degrading these contaminants and inactivating pathogens. Recent advancements in cold plasma have shown significant potential in water treatment applications. Cold plasma has demonstrated efficacy in disrupting bacterial biofilms, reducing antibiotic contaminants, and degrading complex organic pollutants, making it an innovative and eco-friendly alternative to conventional methods. This review examines the principles of plasma technology, evaluates its performance in water sanitation, and addresses the challenges of scalability and economic feasibility. By integrating cold plasma into existing water treatment systems and developing standardized protocols, this technology can significantly contribute to sustainable water management and public health, supporting the Sustainable Development Goals.

**Sustainability Statement**

This review contributes to several United Nations SDGs by offering an innovative and eco-friendly solution to enhance water quality and safety. This technology effectively degrades complex pollutants and inactivates pathogens, thereby improving public health and promoting sustainable water management. It supports SDG 6 by providing access to clean water, SDG 3 by reducing waterborne diseases, and SDG 12 by minimizing the use of harmful chemicals. Additionally, its energy efficiency aids in climate action (SDG 13) and protects aquatic ecosystems (SDG 14). Integrating cold atmospheric plasma into existing water treatment systems encourages technological advancement (SDG 9), while fostering partnerships among academia, industry, and governments amplifies its global impact (SDG 17).

## Introduction

Access to clean water is a fundamental human right, yet over 785 million people lack sufficient drinking water supplies, and ∼2 billion use water sources contaminated with faecal matter, leading to severe health implications, including around 485 000 diarrheal deaths annually (WHO 2020). Traditional water treatment methods such as chlorination, UV irradiation, and filtration, often fall short in addressing the persistence of complex pollutants like dyes, pharmaceuticals, and antibiotics (Abdo et al. 2022, Shi et al. 2022, Hamza and Abd-Elmaksoud 2023). The increasing demand for clean water, driven by rapid industrialization, population growth, climate change, and agricultural expansion, exacerbates water scarcity and pollution, posing significant challenges to public health, environmental integrity, and sustainable development.

Cold atmospheric plasma technology has emerged as a promising method for water sanitation, capable of addressing these complex challenges. Cold plasma generates reactive oxygen and nitrogen species (ROS/RNS) at room temperature, effectively degrading pollutants and inactivating pathogens. This review aims to provide a comprehensive appraisal of cold plasma technology, showcasing its potential in enhancing water quality and promoting sustainable development. The integration of cold plasma technology into existing water treatment frameworks can significantly improve the efficacy of water sanitation processes, reduce environmental pollution, and enhance public health outcomes.

**Figure 1.**
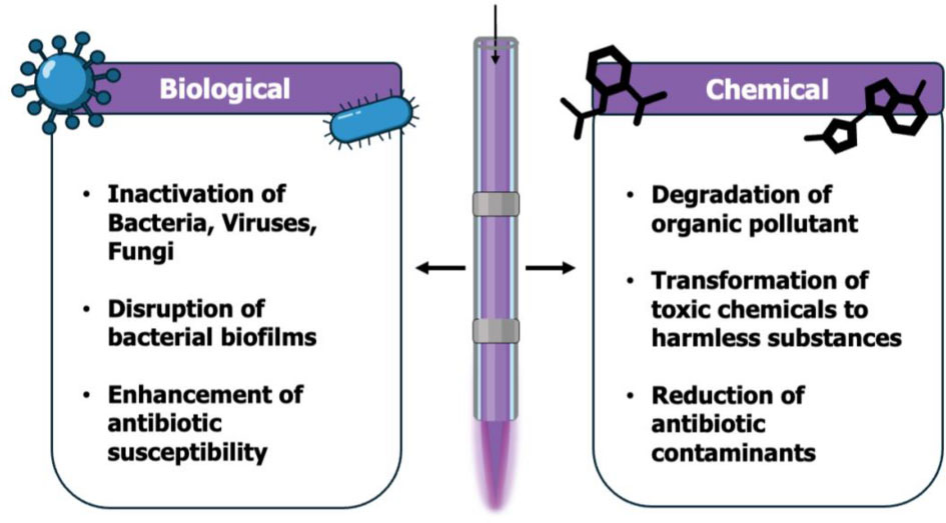
Advantages of cold atmospheric plasma over traditional AOPs in enhancing water quality through its biological and chemical interactions.

Cold plasma technology offers several advantages over traditional advanced oxidation processes (AOPs), including its efficacy in disrupting bacterial biofilms, reducing antibiotic contaminants, and degrading complex organic pollutants. These capabilities make cold plasma an innovative and eco-friendly alternative to conventional water treatment methods. This review examines the principles underlying plasma technology, evaluates its efficacy in water sanitation, discusses its applications across various sectors, and identifies the challenges associated with its scalability and economic feasibility. Figure 1 illustrates the biological and chemical interactions of cold plasma technology, highlighting its advantages over traditional AOPs in enhancing water quality.

### The emergence of cold plasma as a sanitation technology

Cold plasma technology has evolved significantly from its early conceptualization as ‘radiant matter’ in 1879 (Crooks 1879) to the ‘fourth state of matter’ in 1928 (Langmuir 1928) and now its current applications in water sanitation. While initial research focused on plasma’s potential for sterilization in the 1960s, modern advancements in the 20th century have refined plasma, highlighting both its anti-inflammatory and antimicrobial activities (Shekhter et al. 1998, Stoffels et al. 2002, Alkawareek et al. 2012a, Flynn et al. 2015, Brun et al. 2018, Bernhardt et al. 2019). Recent innovations such as the plasma pencil and atmospheric pressure plasma jet demonstrate cold plasma’s capacity to provide precise and localized treatment capabilities ideal for water treatment (Laroussi et al. 2006, Weltmann et al. 2009). These advancements enable cold plasma to address complex pollutants and microbial contamination, making it a useful tool for sustainable water management. Bruggeman et al. (2017) provide an overview of atmospheric pressure non-equilibrium plasmas, emphasizing their unique capabilities in generating reactive species essential for environmental applications, including water treatment​ (Bruggeman et al. 2017).

### Principles and generation of cold atmospheric plasma

Plasmas are categorized into thermal and non-thermal types based on their thermodynamic properties. Thermal plasmas operate at extremely high temperatures (between 10 000 and 25 000 K), while useful for sterilization but unsuitable for biological applications due to the risk of thermal damage (Elsaie and Kammer 2008, Heinlin et al. 2010). In contrast, non-thermal or ‘cold’ plasmas are partially ionized and operate at near room temperatures, allowing them to interact safely and effectively with biological and chemical material, making them ideal for water sanitation, where biological and chemical safety is an important consideration. Types of cold atmospheric plasma include:

- **Dielectric barrier discharge (DBD):** plasma is generated between two electrodes separated by an insulating dielectric barrier when an alternating current is applied. This stable plasma stream can break down pollutants and inactivate pathogens in water. According to Brandenburg (2017), DBD technology ensures consistent plasma generation for stable ROS/RNS production and effective pollutant degradation in water treatment applications​ (Brandenburg 2017). Recent advancements in DBD technology, as outlined by Lu et al. (2023), have led to more stable and uniform plasma generation, further enhancing its scalability and effectiveness for industrial water treatment (Lu et al. 2023).
- **Plasma jet:** uses coaxial electrodes with a feed gas flowing between them. The central electrode is energized, generating a precise plasma stream suitable for targeted water treatment applications. Xiong et al. (2012) demonstrate that APPJs can deliver reactive species through guided ionization waves, enhancing efficacy for localized water treatment​ (Xiong et al. 2012). Moreover, Lu et al. (2014) elaborate on the control of plasma jets, allowing for enhanced distribution of reactive species in non-uniform water systems for effective pollutant degradation (Lu et al. 2014).
- **Air plasma:** utilizes ambient air ionized by electrical discharges, eliminating the need for additional feed gases. This cost-effective method is suitable for large-scale water sanitation.

Plasma’s effectiveness extends far beyond the mere generation of ROS and RNS such as ozone (O_3_), hydrogen peroxide (H_2_O_2_), hydroxyl radicals (•OH), superoxide (O_2_•−), nitric oxide (NO), and peroxynitrite (ONOO−). While these species play a role in the degradation of pollutants and inactivation of pathogens, plasma’s impact is not limited to ROS/RNS. Plasma’s interaction with target surfaces triggers additional reactions, creating secondary reactive species, tailored to the specific environmental through complex biological or chemical milieu in which the plasma operates (Tampieri et al. 2023). Additionally, the physical mechanisms of plasma—such as electric fields, UV radiation, and ionizing particles—provide a treatment approach that cannot be fully replicated by conventional chemical methods (Li et al. 2024).

The combination of chemical and physical mechanisms underpins plasma’s capacity to sanitize water, break down pollutants, and disinfect surfaces in ways that chemical treatments struggle to treat under conventional contact time. Several variables influence plasma treatment effectiveness, including gas type, gas flow rate, water flow rate, voltage, frequency, and exposure mode (Murugesan et al. 2020).

These short-lived and long-lived species play roles in both chemical degradation and microbial sterilization. For chemical degradation, long-lived species are primarily responsible for breaking down pollutants over time. In contrast, short-lived species sterilize due to their immediate oxidative impact on microbial membranes and DNA, often resulting in irreversible cellular damage within seconds of exposure. This combination of short- and long-lived species provides a flexible mode of action for contaminants or pathogens degradation.

### Plasma activated water

Recent advancements in cold plasma technology include the development of plasma activated water (PAW), an innovative and eco-friendly sterilization method. PAW has demonstrated strong antimicrobial properties and the ability to degrade pharmaceuticals, dyes, and pesticides in wastewater, due to the composition and diversity of its reactive species such as O_3_, hydrogen peroxide H_2_O_2_ ozone, and various nitrogen oxides. These reactive species are stable in water and can remain effective for extended periods.

PAW is effective in killing bacteria, viruses, and fungi, making it useful for disinfecting drinking water and treating wastewater. The reactive species in PAW can oxidize and break down complex organic pollutants into less harmful compounds. For instance, recent studies have shown PAW’s effectiveness in degrading steroids (Gao et al. 2022), removing heavy metals (Naz et al. 2021), degrading ibuprofen (Li et al. 2021), removing ammonia nitrogen (Fan et al. 2021), removing bromoamine acid (Xin et al. 2020), removing *p-*chlorophenol (Wang et al. 2020), and degrading textile dyes (Reddy and Subrahmanyam 2012, Tichonovas et al. 2013, Attri et al. 2018). PAW has also proven effective against viruses in wastewater (Guo et al. 2018a, Su et al. 2018, Ghernaout and Elboughdiri 2020, Kaushik et al. 2023, Upadrasta et al. 2023, Zver et al. 2023). PAW demonstrates the capacity to disrupt biofilms due to the high concentration of reactive species, which can penetrate protective matrices and undermine biofilm structural integrity (Shambharkar et al. 2024). This potential improves the management and control of biofilms in water systems (Mai-Prochnow et al. 2021, Xu and Tan 2023).

PAW is being studied for agricultural applications, such as enhancing seed germination (Guragain et al. 2021, 2023, Rathore et al. 2022), and promoting plant growth (Rashid et al. 2021, Than et al. 2022). Post-harvest, PAW can extend the shelf-life of fruits and vegetables by reducing microbial load without negatively affecting sensory properties (Soni et al. 2021, Rothwell et al. 2023). PAW can also be used to wash produce to remove pesticide residues (Sarangapani et al. 2020).

Recent configurations of cold plasma devices use multiphase discharges where plasma gas is bubbled through water, creating ‘plasma microbubbles’. These have been effective for degrading cefixime (Zhang et al. 2021), mixed dye pollutants (Zhou et al. 2021), Per- and Poly-fluoroalkyl substances (Alam et al. 2023), algal cell inactivation (Rao et al. 2023), hexavalent chromium reduction (Ponraj et al. 2023), and biofilm removal in pipes (Xu and Tan 2023). While PAW has shown promise, it is essential to critically assess its limitations, such as the scalability of plasma systems, energy consumption, and the long-term stability of reactive species, which need further investigation.

Biological impact of cold plasma technology in water sanitation

### Antimicrobial effects

Cold plasma technology has demonstrated extensive antimicrobial effects against both Gram-positive and Gram-negative bacteria. Reactive species generated by plasma, such as O_3_, O_2_^−^, OH^−^, NO, and NO_2_ interact with bacterial membrane, causing cell rupture (Jungbauer et al. 2021, Abdel-Wahed et al. 2022). Gram-negative bacteria, which have thinner peptidoglycan cell walls, are generally more susceptible to plasma than Gram-positive bacteria (Mai-Prochnow et al. 2016). These reactive species interact in multiple ways:

- **Oxidation**: ROS, such as hydroxyl radicals (•OH), are potent oxidizers that can cause double-strand breaks in bacterial DNA, induce oxidative stress, and damage essential cellular components.
- **Disruption of microbial cells:** ROS and RNS damage microbial cell membranes, proteins, and DNA through oxidative stress, leading to cell lysis and death (Tabares and Junkar 2021).
- **Acidification**: The production of acidic compounds (e.g. H_2_O_2_, NO_3_−) lowers the pH of the treated water, creating an inhospitable environment for pathogens.
- **Physical effects**: The physical energy produced by plasma—such as UV radiation and electric fields—also plays a crucial role in microbial inactivation and pollutant degradation (Li et al. 2024).

This multi-target approach reduces the risk of developing resistance compared to conventional antimicrobial treatments, as previously demonstrated with *Staphylococcus aureus* and *Escherichia coli* (Matthes et al. 2014, Nicol et al. 2020). Plasma has proven effective in eradicating multidrug-resistant bacteria, including the ESKAPE pathogens (Isbary et al. 2010, Joshi et al. 2011, Alkawareek et al. 2012a, b, 2014, Flynn et al. 2015, 2016, 2019, Brun et al. 2018, Alshraiedeh et al. 2020), and *Clostridium difficile* spores (Claro et al. 2015, Connor et al. 2017), making it a powerful tool in combating antibiotic-resistant infections and improving water sanitation. Furthermore, plasma improves the susceptibility of methicillin-resistant *S. aureus* (MRSA) to several antibiotic classes, including β-lactam (Luhrmann et al. 2016, Guo et al. 2018b).

### Biofilm penetration and disruption

**Figure 2.**
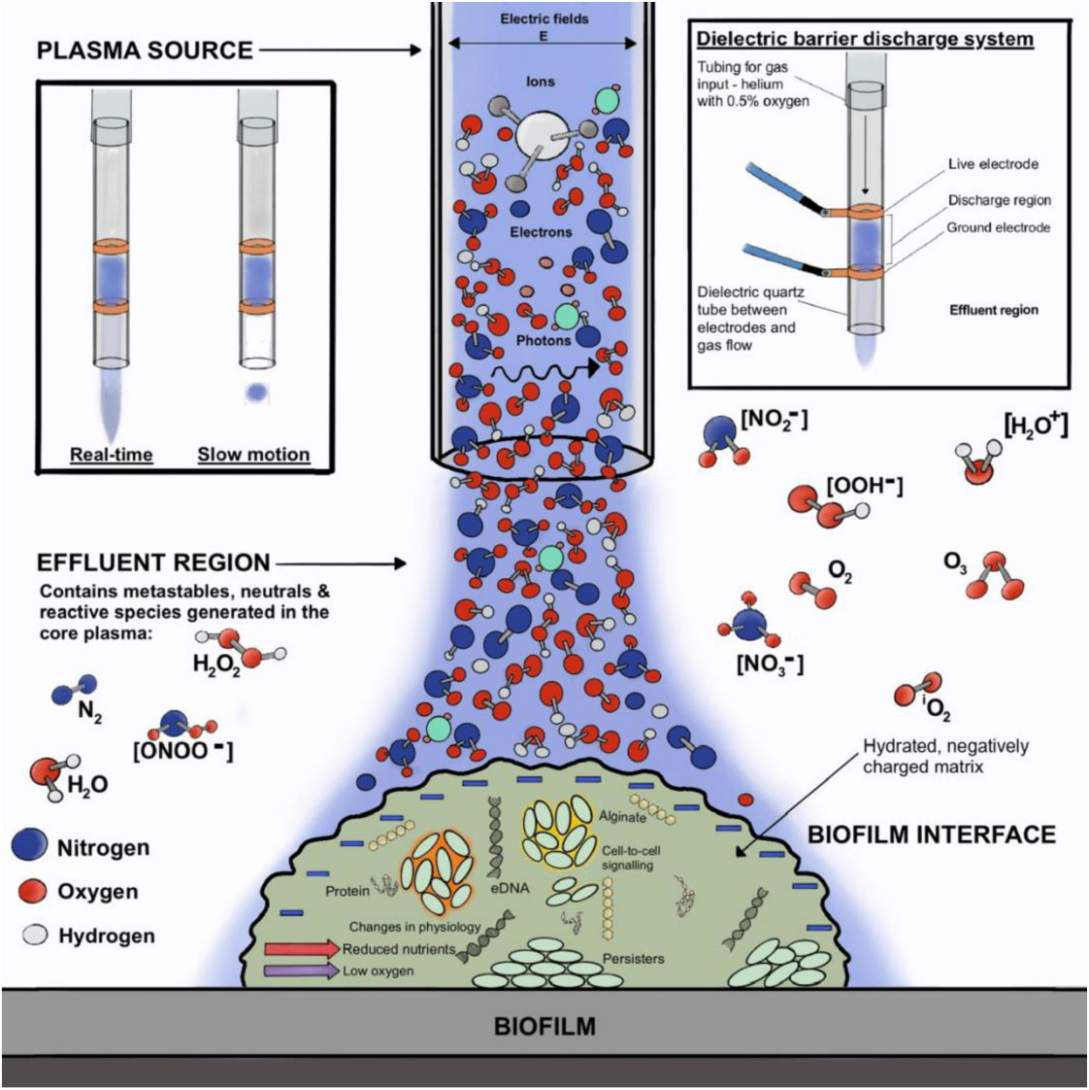
Illustration of the impact of cold plasma on a biofilm depicting major cellular targets and the diverse array of reactive species produced by a cold plasma jet.

Cold plasma can effectively disrupt bacterial biofilms, which express high tolerance against conventional water treatment methods. Biofilms are complex microbial communities encapsulated within a protective extracellular polymeric substances matrix. This matrix, composed of eDNA, proteins, and polysaccharides, imparts considerable tolerance to antimicrobial treatments. Cold plasma produces reactive species, UV radiation, and electric fields, which disrupt the protective biofilm matrix, reducing the biofilm thickness and viability (Ziuzina et al. 2015). Figure 2 provides a schematic overview of the interaction of cold plasma with a bacterial biofilm. Studies have shown that plasma’s effectiveness against *Enterococcus faecalis* biofilms (Theinkom et al. 2019) and food pathogens such as *E. coli* O157: H7 biofilms (Niemira et al. 2018).

A recent review emphasized the potential of cold plasma technologies in developing antiadhesive coatings (Hage et al. 2022). Additionally, cold plasma’s impact on quorum sensing related to biofilms has also been explored, with plasma treatment leading to the downregulation of quorum sensing controlled virulence genes in *P. aeruginosa* (Ziuzina et al. 2015). The role of plasma in reducing virulence in *P. aeruginosa* was further examined, and it was found that plasma could modify lipopolysaccharide and degradation N-acyl homoserine lactone signalling molecules, resulting in attenuation of quorum sensing-dependent virulence (Flynn et al. 2016, Barakat et al. 2019, Maybin et al. 2023).

### Antifungal and antiviral applications

While much of the research on plasma focuses on its bactericidal effects, its potential against fungi and viruses is also gaining attention. The primary mechanisms attributed to this susceptibility include the disruption of fungal cell walls and interference with their metabolic processes. For instance, recent studies have highlighted plasma’s efficacy against dermatophytic fungi (Safi-Samghabadi et al. 2022), *Candida albicans* biofilms (Leite et al. 2021), and *Aspergillus chevalieri* (Molina-Hernandez et al. 2022).

The generation of ROS/RNS from plasma disrupts viral envelopes and hinders their replication processes, offering a broad spectrum against enteric viruses such as norovirus, adenovirus, and hepatitis A (Aboubakr et al. 2016, Chen et al. 2020, Abdel-Wahed et al. 2022), HSV-1 (Bunz et al. 2020), and bacteriophages like MS2, T4, and ɸX174 (Guo et al. 2018a). Importantly, Jin et al. (2021) demonstrated its capacity to significantly reduce the infectivity of SARS-CoV-2-like pseudoviruses (Jin et al. 2021). Furthermore, research by Upadrasta et al. (2023) evaluated the use of plasma activated mist against surrogate viruses for SARS-CoV-2 (Upadrasta et al. 2023). An 80-day clinical trial at Nicosia General Hospital tested cold plasma’s effectiveness in reducing viral loads in COVID-19 wards, and found that plasma devices, especially when integrated with ion emitters, successfully neutralized the virus (Loizou et al. 2022).

Chemical impact of cold plasma in water sanitation

### Plasma degradation of dyes

Traditional wastewater treatment methods such as absorption, adsorption, filtration, chlorination, and microbiological treatment often fail to remove or degrade complex organic pollutants like dyes. These pollutants, which can be mutagenic or carcinogenic, often transition from primary to secondary pollutants (Lutterbeck et al. 2015, Aziz et al. 2017, Mello Souza et al. 2022). Therefore, AOPs are used to treat these organic pollutants in wastewater (Panorel et al. 2013, Lutterbeck et al. 2015, Turkay et al. 2018, Kanjal et al. 2020). AOPs are typically employed to treat these pollutants. However, conventional AOPs, which generate highly oxidizing species such as O^3^/H_2_O_2_, Fe^2+/^H_2_O_2_/UV, and γ-ray/H_2_O_2_, have their limitations. These methods are often not eco-friendly or economically viable due to their reliance on chemicals and energy-intensive processes (Lai et al. 2017, Janssens et al. 2019, Koltsakidou et al. 2019).

Cold plasma technology offers a promising alternative to these traditional methods. By primarily consuming electrical power and avoiding chemical additives, plasma-based AOPs present a minimal environmental impact and enhanced operational efficiency. Operating effectively at room temperature, the ROS and RNS generated by cold plasma degrade complex pollutants into less harmful forms through oxidation, reduction, and radical-mediated fragmentation reactions. Unlike many chemical disinfectants, plasma technology avoids hazardous chemicals, ensuring that no harmful by-products remain. This reduces both environmental damage and health risks associated with chemical disinfectants (Magureanu et al. 2011, Kim et al. 2013, Aziz et al. 2017). Furthermore, plasma technology circumvents issues such as extended contact times, limited shelf lives, and potential hazards to both the environment and human health, offering a safe, efficient, and sustainable alternative (Uwamahoro et al. 2018).

The degradation of organic dyes in wastewater is a critical environmental challenge due to their hazardous nature. Dyes from textile and paper industries are notorious for their mutagenic and carcinogenic properties, posing significant risks to aquatic ecosystems (Rodrigo et al. 2007, Gao et al. 2010). Traditional dye removal methods, such as biological, physicochemical, and chemical treatments, have numerous limitations, especially with complex structures like azo dyes, which resist biodegradation and can produce toxic byproducts (Pandey et al. 2007, Chandanshive et al. 2018). Recent advancements in plasma technology have shown promising results in overcoming these challenges. Table 1 provides an overview of different plasma systems used for organic dye removal, showcasing the target pollutants, degradation efficiency, and treatment times.

**Table 1.** Overview of different types of plasma systems used for organic dye removal.

Plasma source	Target pollutant	Degradation efficiency (%)	Treatment time (min)	Reference
Glow discharge	Acid Blue 25	78	60	Ghodbane et al. (2014)
Glow discharge + TiO_2_	Acid Blue 25	90	60	Ghodbane et al. (2014)
Corona discharge	Acid Blue 25	100	35	El-Tayeb et al. (2016)
Contact glow discharge electrolysis	Acid brilliant red B	94.99	2	Gao et al. (2003)
GAD falling film reactor coupled with TiO_2_	Acid Green 25	94	180	Saïm et al. (2015)
Corona discharge	Acid Orange 142	∼90	90	Fahmy et al. (2020)
Corona discharge	Acid Orange 7	83	45	Li et al. (2007)
Contact glow discharge electrolysis	Acid Orange 7 2	95	90	Jin et al. (2011)
Pulsed discharge plasma coupled with MWCNTs-TiO_2_/δ-Al_2_O_3_	Acid Orange 2	100	60	Li et al. (2016)
DBD	Acid Red 88	95	1	Tang et al. (2009)
DBD	Acid Red 88	92	4	Tang et al. (2009)
Contact glow discharge electrolysis	Cationic Blue SD-GTL	99.7	3	Jin et al. (2010)
DBD	Crystal Violet	100	1	Rahimpour et al. (2019)
DBD	Crystal Violet	90.6	25	Reddy and Subrahmanyam (2012)
RF-Plasma jet	Crystal violet	100	12	Taghvaei et al. (2019)
DBD	Indigo Carmine	99	3.2	Muradia (2013)
Multi-needle configuration	Methyl Orange	∼90	15	Sun et al. (2012)
Gas discharge circulatory airtight configuration	Methyl Orange	92	20	Jiang et al. (2012)
Pulsed electrical discharge assisted with modified activated carbon fibers	Methyl Orange	100	30	Jiang et al. (2013)
DBD	Methyl Orange	91	130	Tao et al. (2016)
Gas discharge circulatory airtight reactor	Methyl Orange	93.7	20	Jiang et al. (2012)
DBD	Methyl Orange	94.1	80	Tao et al. (2016)
Pulsed corona-multi-needle reactor	Methyl Orange	84.3	15	Sun et al. (2012)
DBD	Methyl Red	93	10	Piroi et al. (2009)
Glow discharges generated in contact with flowing liquid cathode	Methyl Red	99	NA	Jamróz et al. (2014)
DBD	Methyl Violet	99	30	Chen et al. (2009)
Corona discharge	Methylene Blue	90	20	Magureanu et al. (2008)
DBD	Methylene Blue	∼98	30	Wu et al. (2019)
Point-to-plate	Methylene Blue	∼100	60	de Brito Benetoli et al. (2012)
Corona discharge	Methylene Blue	∼100	120	Malik et al. (2002)
Double DBD plane-to-plane	Methylene Blue	67.1	40	Wang et al. (2017)
Needle type reactor	Methylene Blue	81.4	20	de Brito Benetoli et al. (2012)
Microplasma bubble	Methylene Blue	>99.9	3	Zhou et al. (2021)
APPJ, using He gas	Methylene Blue	∼100	40	Abdel-Fattah (2019)
APPJ (argon plasma)	Methylene Blue	100	40	Chandana et al. (2015)
Indirect-APPJ	Methylene Blue	71	20	Attri et al. (2018)
Indirect-APPJ	Methyl Orange	81	20	Attri et al. (2018)
Indirect-APPJ	Congo Red	76	20	Attri et al. (2018)
Direct-APPJ	Methylene Blue	95	20	Abdelmalek et al. (2006)
Direct-APPJ	Methyl Orange	97	20	Abdelmalek et al. (2006)
Direct-APPJ	Congo Red	86	20	Abdelmalek et al. (2006)
Microplasma bubble	Acid Orange 2	>99.9	5	Zhou et al. (2021)
Gliding arc	Acid Orange I	95	30	Abdelmalek et al. (2006)
Gliding arc	Crystal Violet	89	60	Abdelmalek et al. (2006)
Gliding arc	Eriochrome Black T	88	60	Abdelmalek et al. (2006)
NTAPP coupled with Cu–CeO_2_ NPs	Reactive Black-5 dye	77.4	25	Pandiyaraj et al. (2021)
Corona discharge	Reactive Brilliant Blue	∼91	60	Ren et al. (2015)
Microplasma array	Remazol Blue	99.4	16	Meiyazhagan et al. (2020)
Corona discharge	Rhodamine B	77	60	Sugiarto et al. (2002)
Corona discharge	Methyl Orange	70	60	Sugiarto et al. (2002)
Corona discharge	Chicago Sky Blue	91	60	Sugiarto et al. (2002)

Most recently, Liu et al. (2024) demonstrated the efficiency of their plasma process in decontaminating wastewater with mixed organic. Their plasma and reactor design significantly improved treatment efficiency by 114.7%, this was attained through a synergistic mechanism involving ROS and enhanced electron transfer, leading to complete dye degradation. Furthermore, *in vivo* zebrafish tests confirmed the treated water’s biosafety, indicating its suitability for reuse in agricultural, industrial, and urban water systems (Liu et al. 2024).

A review by Yin et al. (2023) highlighted that the decolourization rate of dyes depends on several factors, including gas flow rate, working gas composition, duration of the voltage pulses, gap distance, injected power, barrier thickness, and types of electrode materials (Yin et al. 2023). Overall, these findings show the potential of plasma technology in addressing the challenges posed by complex organic pollutants in wastewater, offering a more effective and sustainable solution compared to traditional methods.

### Degradation of pharmaceuticals including antibiotics

Pharmaceuticals are increasingly detected in aquatic environments, presenting significant ecological and public health challenges. Traditional wastewater treatment methods do not remove these compounds completely, resulting in their accumulation in water bodies. Recent advancements in plasma technology, as detailed in Table 2, highlight various plasma systems that target specific pharmaceutical pollutants. The data in this table illustrate the degradation efficiency and treatment times for each pollutant, showcasing the potential of plasma devices to efficiently degrade pharmaceutical residues in wastewater. This comparison emphasizes the diversity in plasma systems’ capabilities, including the effectiveness of different plasma sources (e.g. DBD, corona discharge) in achieving high degradation efficiencies for compounds such as carbamazepine and paracetamol, often in short treatment times.

**Table 2.** Comparison of the degradation of the pharmaceuticals by different plasma systems.

Plasma source	Target pollutant	Degradation efficiency (%)	Treatment time (min)	Reference
DBD	17-β-Estradiol	100	30	Gao et al. (2013)
DBD microbubbles	Aniline	82.7	60	Liu et al. (2018)
DBD	Bisphenol A	96	5	Hijosa-Valsero et al. (2014)
DBD	Carbamazepine	100	5	Liu et al. (2012)
DBD	Carbamazepine	94.00	60	Krause et al. (2011)
Corona	Carbamazepine	98	30	Krause et al. (2009)
Corona	Clofibric Acid	100	30	Krause et al. (2009)
Corona	Iopromide	99	30	Krause et al. (2009)
DBD	Cyanide	99	3	Hijosa-Valsero et al. (2013)
Corona discharge	Diclofenac	100	15	Dobrin et al. (2013)
DBD	Enalapril	90	20	Magureanu et al. (2013)
DBD	Nitrobenzene	Unknown	60	Nawaz et al. (2019)
DBD	Paracetamol	81	60	Baloul et al. (2016)
Corona discharge	Paracetamol	100	20	Panorel et al. (2013)
DBD	Pentoxifylline	92	60	Magureanu et al. (2010)
APPJ	Telmisartan	Unknown	20	Vasu et al. (2019)
DBD	Tributyltin	96	5	Hijosa-Valsero et al. (2014)

Additionally, antibiotic contaminants pose substantial environmental and health risks by contributing to the proliferation of antibiotic-resistant genes. Table 3 demonstrates the efficacy of plasma devices in degrading antibiotics. Notably, plasma treatment significantly reduces the antimicrobial activity of antibiotics without increasing bacterial resistance, suggesting plasma technology could enhance existing wastewater treatment processes (Sarangapani et al. 2019). Wielogorska et al. (2023) further examined the removal of β-lactam antibiotic residues using cold plasma. This study utilized a helium jet at 6 kV, achieving optimal degradation influenced by antibiotic concentration, water pH, and the presence of polar macromolecules. Importantly, no resistance development was noted in *E. coli* exposed to plasma-treated antibiotics (Wielogorska et al. 2023). These studies emphasize the potential of cold plasma as a viable tool in treating a wide range of pharmaceutical and antibiotic compounds in wastewater and align with Sustainable Development Goal 6 (SDG6—Clean Water and Sanitation).

**Table 3.** Overview of Plasma Systems for the degradation of antibiotics.

Plasma source	Antimicrobial	Degradation efficiency (%)	Treatment time (min)	Reference
NSP-DBD with falling liquid film	Amoxicillin	100	10	Magureanu et al. (2011)
DBD	Amoxicillin	>90	10	Magureanu et al. (2011)
DBD	Oxacillin	>90	30	Magureanu et al. (2011)
DBD	Ampicillin	>90	20	Magureanu et al. (2011)
DBD	Ampicillin	100	3	Smith et al. (2018)
Microplasma bubble	Cefixime	94.8	30	Zhang et al. (2021)
DBD	Ciprofloxacin	88	25	Sarangapani et al. (2019)
Plasma needle type	Ciprofloxacin	84.1	24	Hu et al. (2018)
NSP-DBD plane-to-plane	Enrofloxacin	100	20	Aggelopoulos et al. (2022)
DBD	Norfloxacin	97.1	4	Xu et al. (2020)
DBD	Ofloxacin	91	25	Sarangapani et al. (2019)
Corona discharge	Sulfadiazine	∼100	30	Rong and Sun (2014)
Wetted-wall corona	Sulfadiazine	99	27	Rong and Sun (2014)
Corona discharge	Tetracycline	61.9	50	He et al. (2014)
Corona discharge + TiO_2_	Tetracycline	85.1	50	He et al. (2014)
DBD	Tetracycline	92.3	30	Hao et al. (2020)
Corona discharge	Tetracycline	82.64	60	Wang et al. (2018)
DBD	Amoxicillin	50	5	Wielogorska et al. 2023)

### Development and regulatory approvals of plasma devices

The field of plasma medicine has experienced significant technological advancements and crucial regulatory approvals, marking key milestones in translating laboratory research into effective medical applications. For instance, handheld plasma ‘pens’ offer targeted applications that minimize tissue damage while maximizing therapeutic benefits, particularly in wound treatment and tissue regeneration (Kong et al. 2009). Similarly, the atmospheric pressure plasma jet has transitioned from industrial uses to medical applications, providing a non-invasive method for treatments ranging from wound healing to cancer therapy (Laroussi 2020).

Regulatory recognition has kept pace with technological advancements. For example, the Rhytec Portrait® received US FDA approval in 2008 for dermatological applications (Kilmer et al. 2007), highlighting the growing acceptance of plasma technology in clinical settings. Plasma scalpels, such as the Canady Helios Cold Plasma Scalpel and Hybrid Plasma™ Scalpel, offer advantages like reduced bleeding, minimized tissue damage, and enhanced wound healing, underscoring the integration of plasma technology in surgical procedures (Canady et al. 2013).

The kINPen® range by INP Greifswald/neoplas tools GmbH, including the kINPen 09, kINPen 11, and kINPen® MED, represents a significant advancement. The kINPen® MED, introduced in 2013, was the first plasma jet to receive CE certification as a Class IIa medical device, approved for eradicating skin infections and improving wound healing. Its design, featuring a self-contained power supply and a handheld apparatus, makes it practical for clinical settings (Weltmann and von Woedtke 2017, Schönebeck 2018). Other devices such as the DBD PlasmaDerm® (CINOGY GmbH Duderstadt, Germany) (Tiede et al. 2014, Brehmer et al. 2015), and the Adtec SteriPlas (Adtec Plasma Technology, Adtec Europe, Hunslow, UK) have also received certifications for treating chronic and acute wounds, as well as reducing microbial load. These certifications further demonstrate the efficacy and safety of plasma devices in managing various medical conditions (Heinlin et al. 2010, Isbary et al. 2010, 2012, 2013, Arndt et al. 2018, Bernhardt et al. 2019).

### Is cold plasma technology suitable for scale-up?

Cold plasma technology presents significant potential over other AOPs for water sanitation due to its versatility, efficiency, and low-temperature operation. Despite these advantages, one of the challenges is energy consumption. For example, the energy consumption of DBD plasma for carbamazepine removal is reported to be 0.29–1.8 kWh/m^3^, which is significantly lower than other AOPs such as photocatalysis (>100 kWh/m^3^ (Tang et al. 2014). Similarly, plasma-assisted methane reforming is relatively energy-efficient, with catalytic systems consuming as low as 1.64 kWh/m^3^ (Feng et al. 2022), further highlighting cold plasma’s scalability across multiple industrial contexts.

Table 4 demonstrates cold plasma’s lower energy consumption across various pollutants such as benzene and atrazine. In comparison, processes like UV/Fenton-based AOPs (Wei et al. 2011) and photocatalysis (Tang et al. 2014) often consume considerably more energy and generate more hazardous by-products. Furthermore, Gerrity et al. (2010) highlight that cold plasma’s energy efficiency, with electrical energy per order of removal (EEO) values below 10 kWh/m^3^, can be achieved in pilot-scale studies, thereby reinforcing cold plasma’s potential as a scalable solution (Gerrity et al. 2010, Aggelopoulos 2022). Cold plasma produces significantly fewer harmful by-products than other AOPs like photocatalysis, which often involve chemical intermediates that are environmentally hazardous.

**Table 4.** Energy consumption of plasma-based and conventional water treatment technologies.

Treatment method	Energy consumption (EEO) (kWh/m^3^)	Pollutant removed	Reference
DBD plasma	0.29–1.8	Carbamazepine	Gerrity et al. (2010)
Photocatalysis (UV/H_2_O_2_)	>100	Pharmaceuticals, dyes	Tang et al. (2014)
Corona-DBD plasma	5.79	Methylene blue dye	Xiao et al. (2024)
Plasma-ozonation	19.8	BPA	Wardenier et al. (2019)
Plasma-ozonation	4.48	EE2	Wardenier et al. (2019)
DBD plasma	4.0–7.0	Atrazine	Chen et al. (2023)
DBD plasma	3.7 × 10^−3^	Benzene	Fan et al. (2009)
Plasma-assisted methane reforming (non-catalytic)	2.51	Methane	Feng et al. (2022)
Plasma-assisted methane reforming (catalytic)	1.64	Methane	Feng et al. (2022)
DBD plasma (R1—batch reactor)	0.00174	Cyanide	Hijosa-Valsero et al. (2013)
DBD plasma (R2—coaxial thin falling film)	0.1279	Cyanide	Hijosa-Valsero et al. (2013)

However, challenges remain in optimizing energy efficiency when treating complex pollutants. For instance, plasma-ozonation systems, while effective, display higher energy consumption rates (19.8 kWh/m^3^ for BPA), which may limit their industrial viability without further optimizations (Wardenier et al. 2019). However, these systems still outperform many chemical-based AOPs, which often generate hazardous by-products, making plasma a cleaner, safer alternative in wastewater treatment applications (Aziz et al. 2017).

Recent research has focused on integrating plasma into existing practices. Kooshki et al. (2023) demonstrated the use of DBD plasma for treating bio-contaminated wastewater, which was then reused as a green fertilizer. Their plasma device reduced *Staphylococcus epidermidis*and *E. coli* in water by 7-log and enhanced barley germination by over 20% compared to tap water (Kooshki et al. 2023). Another study combined cold plasma with photocatalytic nanoparticles of TiO_2_ and copper-doped TiO_2_, enhancing the degradation of organic pollutants and microbial inactivation in wastewater (Raji et al. 2022). This synergistic approach leverages both technologies’ strengths to achieve higher efficiency, providing a sustainable solution for improving wastewater treatment efficacy and environmental safety.

While advancements have optimized pollutant degradation efficiency and energy consumption, developing an ideal plasma system for widespread industrial application remains challenging. Key factors for successful scaling include enhancing reactive species production, increasing plasma-pollutant contact area, and achieving optimal operational conditions (Gerrity et al. 2010, Aggelopoulos 2022). The energy consumption figures in Table 4 illustrate the progress that has been made, but also point to the variability in energy demand depending on the pollutant and system configuration.

Despite the growing market for cold plasma technology, economic feasibility in resource-limited settings is a critical concern. The global market for cold plasma was estimated at $1.6 billion in 2021 and is projected to reach $3.3 billion by 2026 (Ltd. 2021). Initial investments and ongoing operational costs, including consumables like gases and maintenance, can be substantial. Cold plasma devices consume ∼0.3–0.4 units of electricity over an 8-h operation, with additional costs for argon gas around £2–£2.5 per shift (Kar et al. 2020). Therefore, while cold plasma shows clear energy efficiency advantages, these must be weighed against infrastructure, operational, and maintenance costs, particularly in resource-limited settings. Integrating cold plasma into existing medical and industrial infrastructures requires thorough evaluation of its economic implications and adaptability.

### Conclusions and recommendations

With global water scarcity and pollution posing increasing threats to public health and ecosystems, cold plasma offers a sustainable and innovative solution aligned with the SDGs. The effectiveness of plasma in both chemical degradation and sterilization lies in its multi-faceted approach. The combination of long-lived ROS/RNS species for the gradual oxidation of pollutants and short-lived, highly reactive species for immediate microbial inactivation demonstrates plasma’s adaptability and broad-spectrum efficacy. Additionally, physical mechanisms further enhance its sterilization potential, ensuring that plasma remains a uniquely capable tool in both water treatment and disinfection strategies. Pilot-scale studies have highlighted its energy efficiency, with a lower EEO compared to other AOPs.

Despite these benefits, plasma technology faces challenges, particularly in energy efficiency, economic feasibility, and the lack of standardized evaluation protocols. Aging water infrastructures, especially in developing regions, exacerbate these challenges due to underinvestment and neglect, leading to increased contamination risks and system failures. Scaling plasma technology for larger applications, such as wastewater treatment or hospital disinfection, requires significant technological advancements and financial investments. As the technology matures and more players enter the market, economies of scale might reduce costs, further enhancing its viability.

To facilitate the adoption of cold plasma technology and support SDGs, the following actions are recommended:

**Optimize Energy Efficiency (SDG 7—Affordable and Clean Energy):**

- Develop advanced plasma generation methods that reduce energy consumption.
- Explore the integration of renewable energy sources to power systems.
- Conduct pilot projects to test and refine energy-efficient configurations.

**Enhance Economic Feasibility and Sustainable Implementation (SDG 12—Responsible Consumption and Production):**

- Perform comprehensive cost-benefit analyses to assess initial investments, operational costs, and long-term savings.
- Develop financial models and incentives to support the adoption of plasma technology.

**Standardize Protocols and Evaluation Frameworks (SDG 9—Industry, Innovation, and Infrastructure):**

- Establish benchmarks for reactive species production, pollutant degradation rates, and microbial inactivation efficiency.
- Develop standardized testing protocols to ensure consistent performance across different systems.

**Integrate Cold Plasma with Existing Water Purification Systems (SDG 6—Clean Water and Sanitation):**

- Conduct studies to determine the best methods for incorporating cold plasma into current water treatment infrastructures.
- Develop modular plasma units that can be easily added to existing systems.

**Develop Strategies for Public Engagement and Education (SDG 11—Sustainable Cities and Communities; SDG 17—Partnerships for the Goals):**

- Launch awareness campaigns to educate the public and stakeholders about the benefits and safety of cold plasma technology.
- Establish strategic partnerships among governments, academia, and industry to drive innovation and adoption.

**Conduct Field Trials and Real-World Applications (SDG 3—Good Health and Well-Being):**

- Implement field trials in diverse environments to gather data on the practical performance and benefits of cold plasma technology.
- Monitor and evaluate the impact of cold plasma on water quality and public health in these trials.
- Use findings to refine technology and develop best practices for its deployment.

By addressing these recommendations, cold plasma technology can be further developed and optimized, paving the way for its widespread adoption in water sanitation and contributing to global efforts in ensuring clean and safe water access.

## Data Availability

This a review article, there are no data to make available.
